# A screen for E3 ubiquitination ligases that genetically interact with the adaptor protein Cindr during *Drosophila* eye patterning

**DOI:** 10.1371/journal.pone.0187571

**Published:** 2017-11-08

**Authors:** Kwami F. Ketosugbo, Henry L. Bushnell, Ruth I. Johnson

**Affiliations:** Biology Department, Wesleyan University, Middletown, Connecticut, United States of America; University of Dayton, UNITED STATES

## Abstract

Ubiquitination is a crucial post-translational modification that can target proteins for degradation. The E3 ubiquitin ligases are responsible for recognizing substrate proteins for ubiquitination, hence providing specificity to the process of protein degradation. Here, we describe a genetic modifier screen that identified E3 ligases that modified the rough-eye phenotype generated by expression of *cindr*^*RNAi*^ transgenes during *Drosophila* eye development. In total, we identified 36 E3 ligases, as well as 4 Cullins, that modified the mild *cindr*^*RNA*^ mis-patterning phenotype. This indicates possible roles for these E3s/Cullins in processes that require Cindr function, including cytoskeletal regulation, cell adhesion, cell signaling and cell survival. Three E3 ligases identified in our screen had previously been linked to regulating JNK signaling.

## Introduction

Covalent attachment of ubiquitin to a protein is a post-translational modification that can signal its degradation by the 26S proteasome (reviewed by [1–4]). This process is crucial for the clearance of proteins when no longer needed in a cell. Protein ubiquitination also serves important proteasome-independent roles and has been implicated in signal transduction [5], protein trafficking [6], endocytosis [7], DNA repair [8], transcriptional regulation [9] and histone modification [10]. Given these diverse and important roles, the ubiquitination system can profoundly influence the development and homeostasis of tissues.

Three core classes of enzyme complexes are required for ubiquitination [1–4]. The ubiquitin-activating enzymes (E1s) catalyze conversion of ubiquitin to ubiquitin-adenylate intermediates that are momentarily bound to E1s. The active ubiquitin-adenylate is then transferred to ubiquitin-conjugating enzymes (E2s). Finally, ubiquitin is transferred to target proteins in reactions catalyzed by ubiquitin ligases (E3s) that provide substrate specificity by dictating which target proteins are ubiquitinated.

E3 ligases are characterized according to their domains which catalyze transfer of ubiquitin to target proteins: the HECT (homologous to the E6AP carboxyl terminus) and RING (Really Interesting New Gene) domains [1, 2]. A more elaborate RBR (RING-between-RING) domain characterizes a subclass of E3s. In addition, proteins containing Cullin, U-box, N-recognin, SKP1 and F-box domains contribute to the formation of functional E3 complexes. In recent annotations, 617 putative E3 ligases were identified in the human genome and 80 putative E3 ligases in the yeast *Saccharomyces cerevisiae*, accounting for 1–2% of the proteins encoded in the genomes of these species [11]. E3 ligases occupy a similar percentage of the *Drosophila* genome [12]. Identifying the substrates of these E3 ligases and the cell behaviors for which their functions are crucial will facilitate a comprehensive understanding of the importance of ubiquitination but is a considerable challenge. Determining which E3 ligases are required in specific tissues is an important first step in meeting this challenge.

The *Drosophila* eye neuro-epithelium has been extensively utilized to study cell behaviors and signals that integrate to generate functional epithelia. This tissue is patterned with high precision during development and disruptions to the epithelium are easily detected in the adult eye. Here we describe a genetic modifier screen that identified E3 ligases that genetically interact with Cindr, a conserved scaffold protein that is essential for eye development [13]. Expression of RNAi transgenes that targeted *cindr* generated a sensitized genetic background that could be modified by mutations in E3 ligase loci. The *UAS-cindr*^*RNAi*.*2*.*21*^ transgene was expressed by the driver line *GMR-GAL4* (the genotype of these retinas is abbreviated to *GMR>cindr*^*RNAi2*^ throughout this manuscript). This modestly compromised multiple cell behaviors that require Cindr, including signal transduction, the correct localization of adhesion proteins, and regulated remodeling of the actin cytoskeleton [13–16]. The E3 ligases identified in our screen therefore have potential roles in regulating any of these conserved cell behaviors during the development of the eye epithelium.

## Materials and methods

### *Drosophila* stocks

All stocks used for our modifier screen were obtained from the Bloomington Drosophila Stock Center (Indiana, USA) and are listed in Results. The *GMR-GAL4; UAS-cindr*^*RNAi2*.*21A*^ */ SM5*: *TM6b* line was generated from *UAS-cindr*^*RNAi2*.*21A*^ transgene [13] and the *GMR-GAL4* driver line [17]. In addition, we utilized the following stocks: *Canton-S*, *w*^*1118*^, *UAS-lacZ* and *UAS-puc* (gifts from R. Cagan), and *bsk*^*1*^ (Bloomington stock number BL-3088), *UAS-bsk* (BL-9310), *cbl*^*F165*^ (BL-9676), *nopo*^*excl42*^ (BL-57335), *nopo*^*Z1447*^ (BL-57334), *puc*^*H246*^ (BL-4390), *UAS-slpr*^*WT-HA*^ (BL-58820), *Traf4*^*EY09771*^ (BL-17600), *UAS-Traf6*.*S* (BL-58991) and *Uev1a*^*DG14805*^ (BL-20440).

### Genetic modifier screen

**Fig 1:**
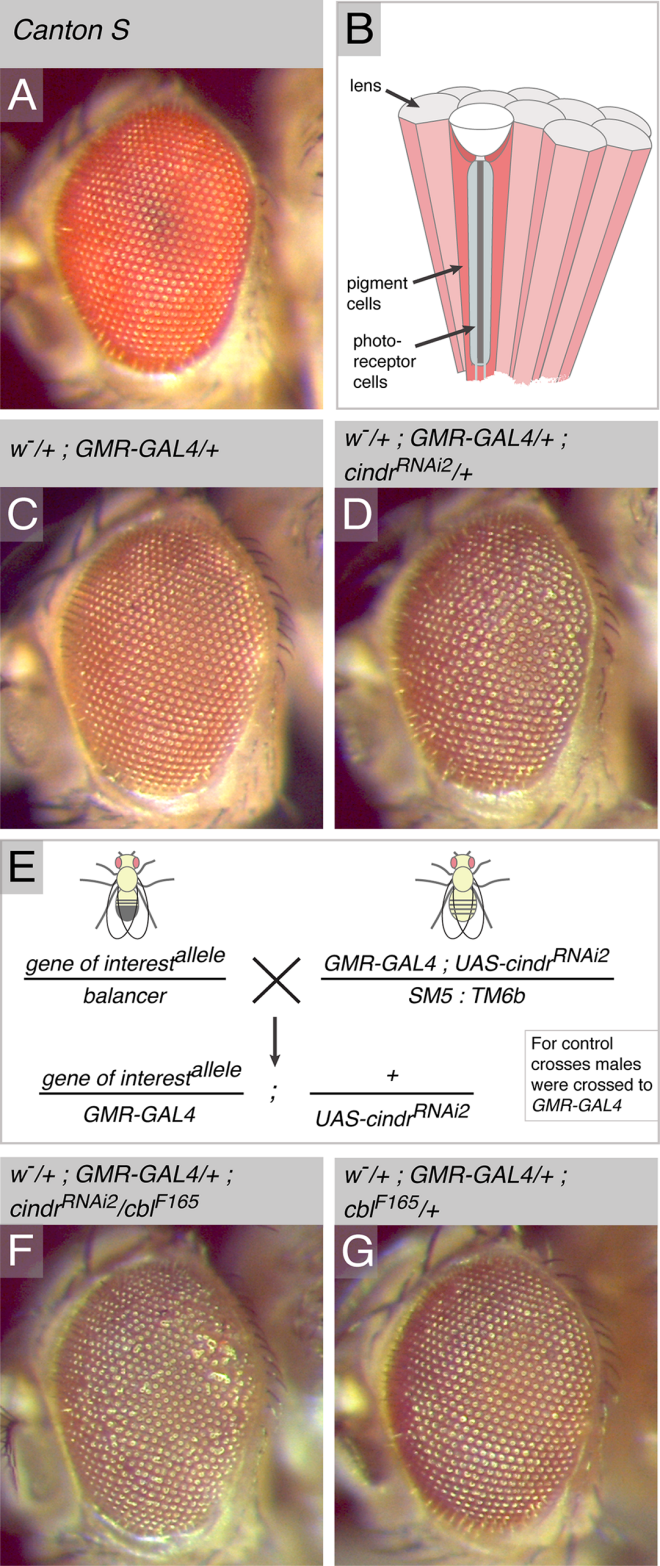
A screen for E3 ligases that regulate *Drosophila* eye development. (A) An eye of the Canton S strain of wild type flies. (B) Cartoon drawing of columnar adult ommatidia. A bundle of photoreceptor cells (grey) forms the core of each ommatidium. These are surrounded by epithelial pigment cells (dark pink). Each ommatidium is capped with a lens (light grey). (C) The eye of an adult heterozygous for *GMR-GAL4*. The eye is wild type in appearance. (D) The eye of an adult heterozygous for *GMR-GAL4* and *UAS-cindr*^*RNAi*^. The eye is mildly mis-patterned. (E) Crossing scheme used in screen. (F) Mis-patterned eye of a fly heterozygous for *cbl*^*F165*^ and expressing *cindr*^*RNAi*^. (G) The correctly-patterned eye of an adult heterozygous for *cbl*^*F165*^ and *GMR-GAL4*.

**Fig 2:**
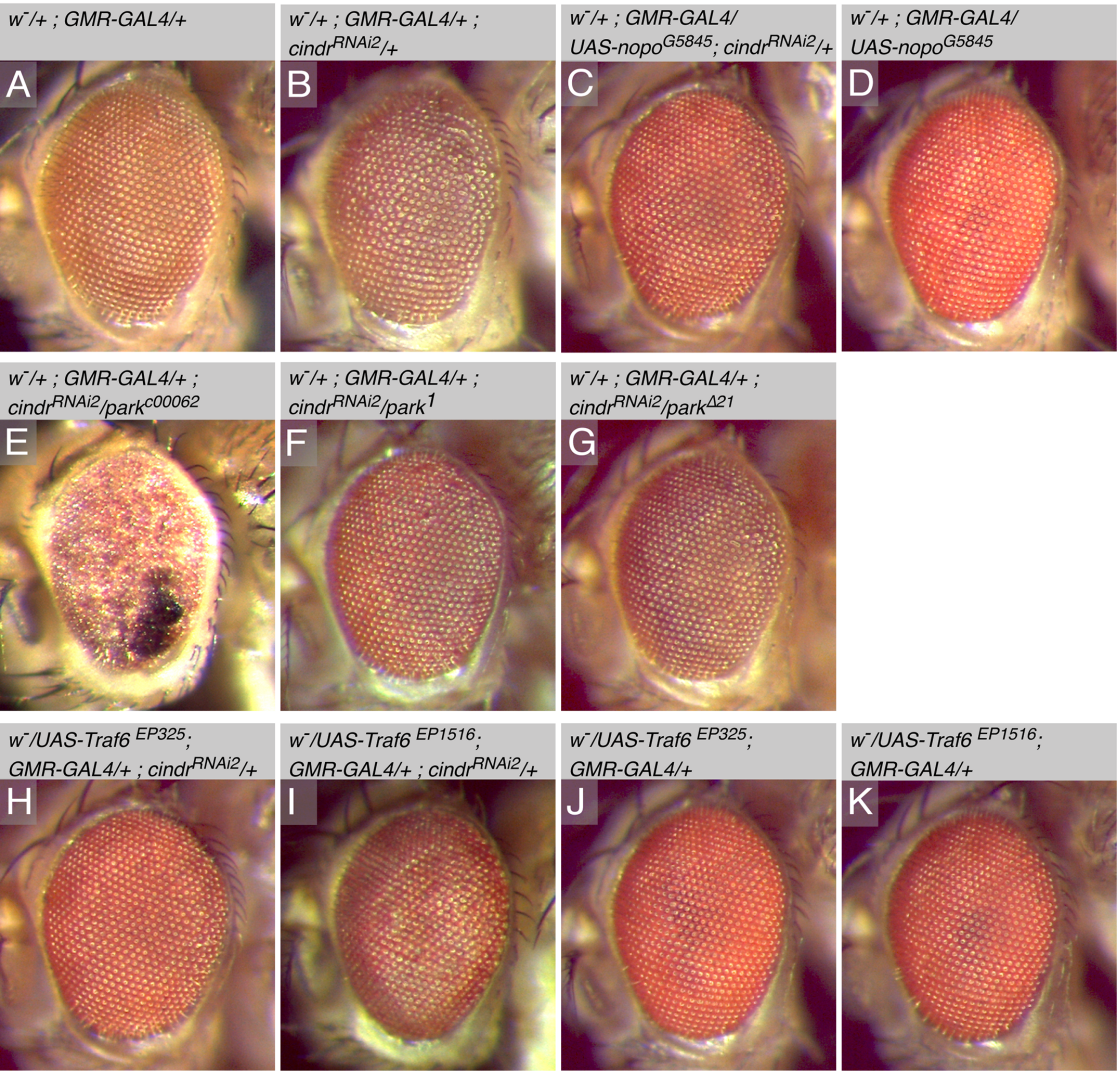
Modification of the rough-eye phenotype by alleles of E3 ligases linked to JNK signaling. (A) A correctly patterned heterozygous *GMR-GAL4/+* eye. (B) Mild mis-patterning manifested as mildly disordered facets that were not arranged in straight rows in the *GMR>cindr*^*RNAi*^ eye. Mis-patterning was suppressed by (C) ectopic *nopo* (*nopo*^*G5845*^) but (D) on its own, *nopo* expression did not disrupt the eye. (E) *park*^*c00062*^ enhanced *cindr*^*RNAi*^ mis-patterning whilst (F) *park*^*1*^ and (G) *park*^Δ21^ suppressed *cindr*^*RNAi*^ mis-patterning. (H) *Traf6*^*EP325*^ and (I) *Traf6*^*EP1516*^ also modestly suppressed the *cindr*^*RNAi*^ rough eye. (J) *Traf6*^*EP325*^ and (K) *Traf6*^*EP1516*^ did not disrupt the eye when crossed to *GMR-GAL4*.

**Fig 3:**
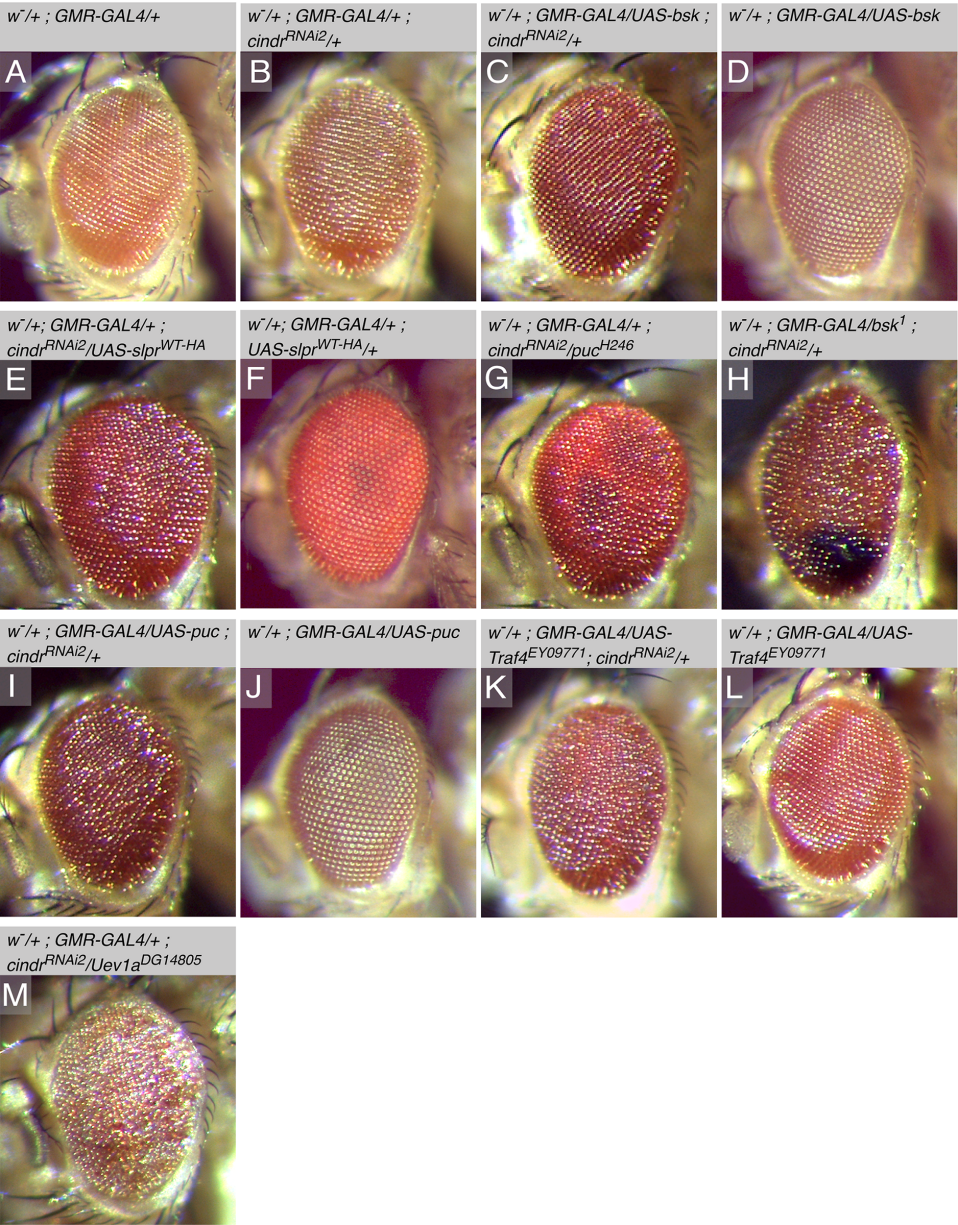
The *cindr*^*RNAi*^ eye is modified by JNK activity. (A) Eye of a *GMR-GAL4* heterozygote and (B) *GMR>cindr*^*RNAi*^ adult. (C) *cindr*^*RNAi*^–induced mis-patterning was mildly enhanced by ectopic *bsk* (D) but (D) on its own *bsk* expression did not disrupt the eye. Similarly (E) ectopic *slpr* enhanced the *cindr*^*RNAi*^ rough eye but (F) the *GMR>slpr* adult eye was correctly formed. (G) *puc*^*H246*^, (H) *bsk*^*1*^ and (I) ectopic *puc* enhanced *cindr*^*RNAi*^- mis-patterning, whilst (J) expression of only *puc* did not perturb patterning. Similarly (K) *Traf4*^*EY0977*1^ enhanced the *GMR>cindr*^*RNAi*^ rough eye whilst (L) *GMR>Traf4*^*EY09771*^ adults had correctly patterned eyes. (M) *Uev1a*^*DG14805*^ severely enhanced the *GMR>cindr*^*RNAi*^ rough eye.

Between six and eight young male flies of each stock screened were crossed to eight to ten virgin *GMR-GAL4; UAS-cindr*^*RNAi2*.*21A*^ */ SM5*: *TM6b* females. For control crosses, males were crossed to *GMR-GAL4* virgin females. Crosses were maintained at 25°C. The parental flies were removed from vials on day seven. On day fourteen the F1 progeny that had emerged were scored blind and independently by two researchers. Scoring was repeated if their assessments differed. Adults were frozen rapidly at -70°C and imaged using a Leica M125 stereo-dissecting microscope fitted with an LED5000HDI ring light and diffuser (data presented in Fig 1 and Fig 2A–2D and 2F–2K and Fig 3D, 3F and 3J) or gooseneck light sources (Fig 2E and Fig 3A–3C, 3E, 3G–3I and 3K–3M), Leica IC80HD camera and Leica Acquire version 3.3 software (Leica Microsystem, Exton, PA). Images were processed using Adobe Photoshop CC (Adobe, San Jose, CA).

### Pupal eye dissection, immunofluorescence and analyses

All crosses and pupae were maintained at 25°C. Eye-brain complexes were dissected at 40 hours after puparium formation (APF) in PBS, fixed on ice in 4% formaldehyde in PBS for 35 minutes, and incubated overnight in rat anti-Drosophila Ecadherin (1:20, DSHB DCAD2) at 4°C. Tissue was then incubated in goat anti-rat secondary antibodies conjugated to AlexaFluor 488 (Jackson ImmunoResearch). Retina were removed from the brain complexes and imaged using a Leica TCS SP5 DM fluorescent microscope and associated LAS AF Software (Leica Microsystem, Exton, PA). Images were processed using Adobe Photoshop CC (Adobe, San Jose, CA). Patterning errors were quantified as previously described [18].

## Results

### Selection of E3 ligases

We explored the *Drosophila melanogaster* genome using the Gene Ontology search function hosted by FlyBase (the database for *Drosophila* Genes and Genomes) to identify loci annotated to have domains or properties associated with ubiquitin ligase activity. These searches generated an initial candidate list of 156 predicted or experimentally confirmed E3 ligases (S1 Table, summarized in Table 1), which included all ubiquitin ligases also identified by FlyBase curators [19–21]. Since Cullin proteins function as scaffolds to assemble E3 ligase complexes [22], we also included the six *Drosophila* Cullins into our candidate list. We did not include F-box and SKP1 proteins, which are components of the Cullin-based E3 complexes.

**Table 1:** Summary of E3 ligase loci and Cullins identified and included in screen.

Class of protein	Number of genes in *Drosophila* genome	Number tested in screen
Golliath	2	0
HECT	14	6
IAP	2	0
RING	119	44
RBR	5	5
ROC	3	3
TRIM	5	3
U-box	6	5
Cullin	6	6
Total	162	72

Our primary goal was to identify E3 ligases that function in the cytosol during epithelial tissue development, since Cindr resides in this compartment. Therefore, we removed from our candidate list all E3 ligases that, at the time, were known or predicted to function primarily in the nucleus, mitochondria, peroxisomes, the endoplasmic reticulum, lysosome or the endosome (see S1 Table). Next, we removed E3 ligases that (at the time) were known to modify Notch signaling (Bre1, Deltex, Mind Bomb 1, Suppressor of deltex, Nedd4, Neuralized) and Decapentaplegic/SMAD signaling (Smurf/Lack), because these signaling pathways are essential for processes that also require Cindr during *Drosophila* eye development [23, 24]. However, we included Cbl, a proto-oncogene that modifies Receptor Tyrosine Kinase (RTK) signaling, including Epidermal Growth Factor Receptor Signaling (EGFR) which is extensively utilized during *Drosophila* eye development [25, 26]. Since the vertebrate orthologs of Cbl and Cindr are known to interact [27], we predicted that alleles of *cbl* would be identified in our screen, which would verify the efficacy of our approach.

The Bloomington Drosophila Stock Center (BDSC) maintains stocks carrying classical mutations or transposable element insertions. Gene expression is modified in many of these *Drosophila* lines. Unfortunately, alleles were not available for 39 of the 110 loci that we wished to screen (S1 Table). Alleles for the remaining 71 loci were obtained.

### The genetic modifier screen

The *Drosophila* eye is a simple neuro-epithelium composed of ~750 hexagonally shaped ommatidia, each capped with a domed lens that is easily observed using a standard stereo-dissecting microscope (Fig 1A and 1B) [28]. Each ommatidium is composed of eight photoreceptor neurons encapsulated by four cone-cells and two primary pigment cells that secrete the material that becomes each lens. Secondary and tertiary pigment cells surround each ommatidium. The hexagonally-shaped ommatidia are neatly packed in rows, giving the adult eye its precisely-ordered appearance (Fig 1A and 1C). Even small disturbances during eye development can disrupt this simple pattern, alter the shape or dimensions of the ommatidial lenses, and give rise to mis-patterned ‘rough’ eyes. Expression of RNAi transgenes that targeted *cindr* generated a mild rough-eye phenotype (Fig 1D). Cindr is required for the regulation of a multitude of cellular processes during eye development including the correct remodeling of the actin cytoskeleton, the appropriate spatio-temporal localization of adhesion proteins, cell-signaling, and the apoptotic removal of superfluous cells from the eye field [13–16]. Given these diverse cell behaviors, the *GMR>cindr*^*RNAi2*^ rough-eye provided a sensitized background for a modifier screen to identify E3 ligases and Cullins essential for eye patterning. Enhancement or suppression of the *GMR>cindr*^*RNAi2*^ eye phenotype could be easily scored.

Males of each candidate stock were crossed to *GMR-GAL4; UAS-cindr*^*RNAi2*.*21A*^ */ SM5*: *TM6b* (abbreviated to *GMR>cindr*^*RNAi*^) or *GMR-GAL4* females and the eyes of adult progeny scored (Fig 1E). All progeny from control crosses had correctly patterned eyes (an example is shown in Fig 1G). As expected, the *cbl*^*F165*^ allele enhanced the *cindr*^*RNAi*^-rough eye (Fig 1F). Alleles of 2 HECT E3 ligase loci, 29 RING E3 loci, 2 RBR loci, 2 TRIM loci and 1 U-box loci modified the *GMR>cindr*^*RNAi2*^ phenotype (Table 2). Alleles of 4 of the 6 Cullins modified *GMR>cindr*^*RNAi2*^ mis-patterning. In many instances, p-element insertions that included UAS sites which potentially increased protein expression and transgenic insertions that disrupted gene loci inversely modified the *GMR>cindr*^*RNAi2*^ adult eye. For example, *Cul2*^*02074*^ reduced mis-patterning whilst this was enhanced by the UAS insertion *Cul2*^*EY09124*^. Similarly, *Ltn1*^*1*^ enhanced mis-patterning whilst the UAS insert *Ltn1*^*G9156*^ suppressed the *GMR>cindr*^*RNAi2*^ phenotype.

**Table 2:** List of E3 ligase and Cullin proteins tested in screen.

Known/ predicted domain class^1^^.^	CG number	Gene symbol	Allele	Nature of allele (known or predicted)^2^^.^	Phenotype: enhanced (E), suppressed (S) or no modification (NM) of *GMR>cindr*^*RNAi2*^
Cullin	CG11261		*c06238*	P insert	E
Cullin	CG11861	*Cul3*	*gft2*	loss of function	S
Cullin	CG1401	*Cul5*	*EY00051*	UAS insert	NM
			*EY21463*	UAS insert	NM
Cullin	CG1512	*Cul2*	*02074*	P insert	S
			*EY09124*	UAS insert	E
Cullin	CG1877	*Cul1*	*BG02329*	GAL4 insert	E
			*EY11668*	UAS insert	NM
Cullin	CG8711	*Cul4*	*KG02900*	P insert	NM
HECT	CG4238		*MI13092*	Minos insert	NM
			*KG04649*	P insert	E
HECT	CG42574	*ctrip*	*G19129*	UAS insert	NM
			*HP35916*	UAS insert	NM
HECT	CG6190	*Ube3a*	*EP3214*	UAS insert	NM
HECT	CG8184		*B*	uncharacterized point mutation	NM
HECT	CG9153	*Sherpa*	*G5486*	hypomorph	S
HECT	CG9484	*hyd*	*15*	uncharacterized EMS mutation	NM
			*c017*	P insert	NM
RING	CG10263	*Hakai*	*KG01389*	P insert	E
RING	CG10523	*park*	*UAS-park*.*G*	UAS line	S
			*c00062*	P insert	E
			*1*	P insert	S
			*∆21*	loss of function	S
RING	CG10916		*f03629*	P insert	E
RING	CG10961	*Traf6*	*EP325*	UAS insert	S
			*EP1516*	UAS insert	S
RING	CG10981	*dgrn*	*DK*	partial loss of function	E
			*EY09862*	UAS insert	NM
RING	CG11329	*Nse1*	*k00605a*	P insert	S
RING	CG12477		*BG01986*	P insert	E
RING	CG12489	*dnr1*	*KG01493*	P insert	NM
			*BG02050*	P insert	NM
RING	CG13025		*EY10081*	UAS insert	NM
	CG13025		*e03112*	P insert	NM
RING	CG13030	*sinah*	*1*	loss of function	S
RING	CG13344		*c05454*	P insert	NM
RING	CG13605		*G14745*	UAS insert	NM
RING	CG14472	*poe*	*01659*	P insert	E
RING	CG15104	*Topors*	*f05115*	amorph	E
RING	CG15141		*KG06005*	P insert	NM
RING	CG15439		*EY01496*	UAS insert	NM
RING	CG16807	*roq*	*EY09493*	UAS insert	E
RING	CG16947		*MI07089*	Minos insert	S
RING	CG17492	*mib2*	*4*	uncharacterized EMS mutation	NM
			*1*	amorph	NM
RING	CG17721		*G18680*	UAS insert	S
RING	CG1815		*EY01163*	UAS insert	NM
RING	CG1909		*C024*	P insert	E
RING	CG32210	*Ltn1*	*1*	not known	E
			*G9156*	UAS insert	S
RING	CG32369		*EY10338*	UAS insert	S
			*MI02469*	Minos insert	NM
RING	CG32486		*CC00904*	P insert	E
			*SH095*	P insert	NM
RING	CG32581		*G10126*	UAS insert	NM
RING	CG32592	*hiw*	*ND8*	loss of function	NM
RING	CG4080		*KG08382*	P insert	NM
			*EY01375*	UAS insert	NM
RING	CG4909	*POSH*	*k15815*	P insert, amorph	NM
RING	CG5140	*nopo*	*G5845*	UAS insert	S
RING	CG5555		*EY00181*	UAS insert	S
RING	CG6752		*c06604*	P insert	E
RING	CG6923		*G4352*	UAS insert	S
RING	CG7037	*Cbl*	*F165*	loss of function	E
RING	CG7184	*Mkrn1*	*EY14602*	UAS insert	NM
RING	CG7376		*e02832*	P insert	E
RING	CG7694		*07551*	P insert ^3^^.^	E
RING	CG8786		*EY09040*	UAS insert	S
RING	CG8910		*c01167*	P insert	NM
RING	CG8974		*G757*	UAS insert	E
RING	CG9086	*Ubr1*	*BG01122*	GAL4 insert	NM
RING	CG9381	*mura*	*EP-643*	UAS insert	E
			*EY00506*	UAS insert	NM
RING	CG9941		*G242*	UAS insert	E
RING	CG9949	*sina*	*SH*	deletion	E
			*3*	amorph	NM
RING-between-RING	CG11321	*LUBEL*	*MB00197*	GAL4 insert	NM
RING-between-RING	CG12362		*MI06577*	Minos insert	E
RING-between-RING	CG33144		*KG08822*	P insert	NM
RING-between-RING	CG5659	*ari-1*	*EY01960*	UAS insert	S
			*EP317*	UAS insert	S
			*EY08909*	UAS insert	NM
RING-between-RING	CG5709	*ari-2*	*07768*	P insert	NM
ROC	CG16982	*Roc1a*	*G824*	UAS insert	NM
ROC	CG16988	*Roc1b*	*dc3*	loss of function	NM
ROC	CG8998	*Roc2*	*KG07982*	UAS insert	NM
			*EP2487*	UAS insert	NM
TRIM	CG15105	*tn*	*f02741*	P insert	E
TRIM	CG31721	*Trim9*	*KG05017*	P insert	NM
TRIM	CG8419		*MB06410*	Minos GAL4 insert	E
U-box	CG2218		*EY02167*	UAS insert	NM
U-box	CG5519	*Prp19*	*G3080*	UAS insert	S
			*07838*	P insert	S
U-box	CG6179		*f08025*	P insert	NM
U-box	CG7747		*f02221*	P insert	NM
U-box	CG9934		*G13471*	UAS insert	NM

1. Classification according to http://flybase.org/reports/FBgg0000069.html, http://flybase.org/reports/FBgg0000128.html, and http://flybase.org/reports/FBgg0000131.html

2. P-element insertions that include UAS sites are listed here as UAS inserts. These may lead to ectopic protein expression in the presence of the *GMR-GAL4* driver, although the UAS insertion may also perturb the locus.

All other P-element insertions are listed as P-inserts. These, as well as the Minos-transposon (Minos insert) lines listed, may display perturbed gene expression.

P-element insertions that include the GAL4 transgene are listed as GAL4-inserts. These may perturb expression of a locus and in addition drive additional expression of the *UAS-cindr*^*RNAi*^ transgene.

3. The 07551 P-element insertion may perturb both *CG7694* and the neighboring locus *fray*.

Alleles of *Cul1*, *Cul2*, *Cul3* and *CG11261* modified the *GMR>cindr*^*RNAi2*^ eye. Diverse roles for *Drosophila* Cul1 and Cul3 have been suggested that may account for their interactions with *GMR>cindr*^*RNAi2*^. Cul1 has been implicated in regulating Cyclin E to promote cell division [29] and may therefore modify mitosis during larval eye development. Cul1 and Cul3 are also regulators of Cubitus Interruptus [30–32], transcription factor activated by Hedgehog signaling, which is required during early eye patterning [23]. In addition, Cul3 has been described as a regulator of the actin cytoskeleton [33–36], though this role has not been explored in epithelia. Cul2 function has been mainly explored in *Drosophila* germline development [37, 38].

Many of the E3 ligases that modified *GMR>cindr*^*RNAi2*^ have yet to be characterized and named (Table 2). In addition, we identified E3 ligases that have been linked to differentiation, signaling, and the modification or maintenance of cell structure or organization. These included *Prp19* which regulates RAS/MAPK signaling [39, 40]; *poe* a component of the spliceosome complex that was recently implicated in regulating MAPK stability [41]; *dgrn*, an antagonist of Notch signaling [42]; *Sherpa*, which is required for Toll signaling [43]; *roq*, which plays a role in mRNA degradation [44]; *Ltn1*, which associates with the ribosome to mediate degradation of polypeptides translated from mRNAs lacking stop codons [45]; *Topors*, which has been implicated in chromatin organization and nuclear lamin organization [46–48]; *mura*, which has also been isolated in screens for loci involved in the DNA damage response, ethanol tolerance, memory and cardiovascular development [49–53]; *ari*-1, which has been implicated in axon pruning and re-wiring and adult myogenesis and is especially important during metamorphosis as it targets the ecdysone receptor [54–57]; *tn*, which is crucial for the assembly and maintenance of myofibrils [58, 59]; and *sinah* and *sina*, which have been implicated in photoreceptor and bristle differentiation [60]. In addition, several E3 ligases connected to Jun-N-terminal Kinase (JNK) signaling modified *GMR>cindr*^*RNAi2*^ phenotypes.

### A cohort of JNK-associated E3 ligases were identified in the screen

A set of sequentially-activated kinases comprise the core of the JNK signaling pathway, a developmentally regulated pathway that is also activated in response to stress signals [61–63]. JNK activity influences the establishment of planar polarity in the fly retina [64], but otherwise does not contribute significantly to eye development [65]. However, we found that a UAS-insertion allele of *no poles* (*nopo*) suppressed *GMR>cindr*^*RNAi2*^ mis-patterning (Fig 2C). Nopo promotes apoptosis in response to Eiger-TNF Receptor signaling [66]. Modification of *GMR>cindr*^*RNAi2*^ by alleles of *parkin* (*park*), was inconsistent: defects were suppressed by *park*^*1*^ and mildly repressed by *park*^*Δ21*^ but severely enhanced by *park*^*c00062*^ (Fig 2E–2G). It is possible that the *park*^*c00062*^ line contains additional mutations that contribute to eye disruption. Parkin has been implicated in inhibiting JNK activity to suppress apoptosis, possibly by indirectly reducing *bsk* expression [67, 68]. Finally, two alleles of *tumor necrosis factor receptor-associated factor 6* (*Traf6*) that potentially drive ectopic *Traf6*, *Traf6*^*EP325*^ and *Traf6*^*EP1516*^ suppressed *cindr*^*RNAi*^ mis-patterning (Fig 2H and 2I). Traf6 promotes JNK activity downstream of the TNF Receptor [69].

Identifying alleles of *nopo*, *parkin* and *Traf6* in our screen suggested that JNK activity is modified in the eye epithelium in response to expression of *UAS-cindr*^*RNAi2*^ transgenes, a relationship that we have observed in *Drosophila* wing epithelia [16]. To verify this, we tested whether alleles of other JNK signaling components modified the *GMR>cindr*^*RNAi2*^ adult eye phenotype. Over-expression of *basket* (*bsk*, which encodes the *Drosophila* JNK [65]) mildly enhanced *cindr*^*RNAi2*^ mis-patterning (Fig 3C). In addition, ectopic *slpr*, a JNKKK that functions upstream of Bsk, [70, 71], also enhanced the *cindr*^*RNAi2*^ rough eye (Fig 3E) as did mutations in the Bsk inactivator, *puckered* (*puc*, [72], Fig 3G). As the potential for JNK activity would have been enhanced in these three genetic manipulations, we expected that manipulations that decreased JNK activity would suppress *GMR>cindr*^*RNAi2*^ adult eye phenotypes. However, mutations in *bsk* and over-expression of *puc* enhanced *cindr*^*RNAi2*^ mis-patterning (Fig 3H and 3I). These data could reflect cross-talk between JNK and other signals that are utilized during eye development, including Notch, Hedgehog and RTK networks [73]. In addition, ectopic *Traf4*, which promotes JNK signaling [74], also enhanced the *cindr*^*RNAi2*^ rough eye as did mutations in the *ubiquitin-conjugating enzyme variant 1a* (*dUev1A)* an E2 enzyme that promotes JNK activity [75] (Fig 3K and 3M). Interestingly, alleles of two Cullins tested in our screen—Cul1 and Cul3—also modified the *GMR>cindr*^*RNAi2*^ adult eye (Table 2). Activity of these Cullins has not been linked to JNK signaling, but they have been implicated in dendrite morphogenesis and apoptosis, processes that require JNK activity. Surprisingly, Cul4, which has been shown to regulate JNK (as well as Wingless) activity [76], did not modify the *GMR>cindr*^*RNAi*^ adult eye–it is possible that the *Cul4*^*KG02900*^ allele used in the screen does not significantly perturb the locus.

**Fig 4:**
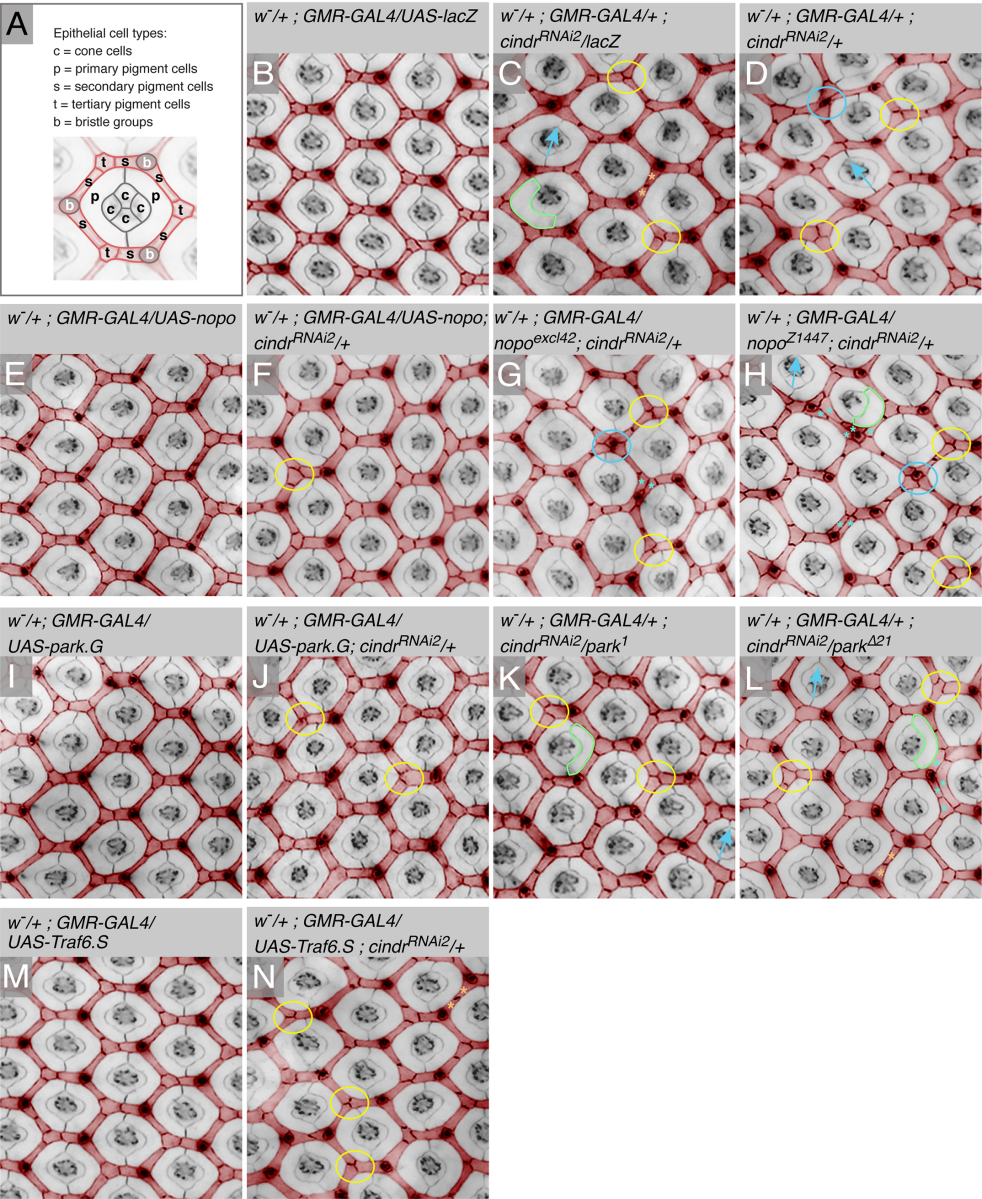
Patterning of the pupal retinal was modified by interactions between *park*, *nopo* and *Traf6* and *cindr*. (A) A single ommatidium in a wild-type eye dissected at 40 h APF, with constituent cell types indicated. Photoreceptors are positioned beneath the surface of the tissue and not clearly observed in this image of the apical eye surface. (B) Small region of a correctly patterned control pupal retina and (C) retina expressing *cindr*^*RNAi*^ together with *lacZ* or (D) only *cindr*^*RNAi*^. Expression of (E) *nopo*^*G5845*^, (F) *nopo*^*G5845*^ and *cindr*^*RNAi*^. (G) Expression of *cindr*^*RNAi*^ in a *nopo*^*excl42*^ heterozygote and (H) *nopo*^*Z1447*^ heterozygote. Expression of (I) *park*, (J) *park* and *cindr*^*RNAi*^. (K) Expression of *cindr*^*RNAi*^ in a *park*^*1*^ heterozygote and (L) *park*^Δ21^, heterozygote. (M) Expression of *Traf6*^*S*^ and (N) *Traf6*^*S*^ and *cindr*^*RNAi*^. Anti-ECad was used to visualize all adherens junctions of retinas. Fluorescence images have been transformed into greyscale and interommatidial cells pseudo-colored red in order to highlight the honeycomb lattice. Examples of patterning defects are indicated as follows: blue arrow = mis-orientation of ommatidial core; outlined in green = small primary pigment cells; yellow circle = tertiary position not defined; blue circle = bristle misplaced and star-like arrangement of cells around bristle; orange asterisks = two cells rather than one in a secondary pigment cell position; blue asterisks = cells grouped in multiple rows rather than single file.

To better understand how *nopo*, *park* and *Traf6* modified the phenotype of *GMR>cindr*^*RNAi2*^ adult eyes, we dissected retinas from pupae in which these E3 ligases were modified (Fig 4). At 40 hours after puparium formation (APF), the stereotyped arrangement of ommatidial cells and the interweaving cell lattice is established (Fig 4A and 4B). Specifically, four cone cells and two primary pigment cells encapsulate each group of photoreceptors, which are buried below the apical epithelium surface. Three bristle groups, three tertiary pigment cells and six secondary pigment cells are arranged about each ommatidium, thus generating the honeycomb lattice (Fig 4A and 4B). This precise cellular pattern is reflected in the arrangement and shapes of the lenses of adult eyes (Fig 1A and 1B). The neat arrangement of interommatidial cells was mildly disrupted in *GMR>cindr*^*RNAi2*^ retinas (Fig 4C and 4D) due to the introduction of a variety of patterning errors which are quantified in Table 3. Specifically, many cells failed to adopt correct positions and shapes and consequently the honeycomb interommatidial cell lattice was mildly distorted. Expression of *nopo*, *park* and *Traf6* generated few defects in the arrangement of cells in the retina (Fig 4E, 4I and 4M, Table 3). However, ectopic *nopo* and *park* significantly restored patterning of interommatidial cells in *GMR>cindr*^*RNAi2*^ retina (Fig 4F and 4J, compare to panel C, Table 3). To verify the contribution of *Traf6* we obtained an additional *UAS* line (BL-58991), which mildly improved patterning of the pupal eye (Fig 4N, compare to 4C, Table 3). Mis-patterning of the lattice of *GMR>cindr*^*RNAi2*^ retinas was mildly enhanced by alleles of *nopo* (Fig 4G, 4H, compare to 4D) and improved by *park*^*1*^ and *park*^*Δ21*^(Fig 4K and 4L, Table 3).

**Table 3:** Quantification of patterning defects in retinas dissected at 40 h APF.

GENOTYPE	Number of defects per hexagonal data point ^1^^.^										Total number of interommatidial cells ^7^^.^		Mis-patterning Score ^8^^.^			SIGNIFICANCE (p-value) ^9^^.^
	cone cell defects ^2^^.^		primary cell defects ^3^^.^		ommatidial orientation defects ^4^^.^		bristle defects ^5^^.^		tertiary defects ^6^^.^							
	Mean	SD	Mean	SD	Mean	SD	Mean	SD	Mean	SD	Mean	SD	Mean	SD	SE	
**Group 1:**	** **	** **	** **	** **	** **	** **	** **	** **	** **	** **	** **	** **			** **	
*w*^*1118*^*/+; GMR-GAL4/+*	**0.00**	0.00	**0.00**	0.00	**0.00**	0.00	**0.09**	0.29	**0.05**	0.23	**11.97**	0.33	**0.26**	0.68	0.08	-
*w*^*1118*^*/+; GMR-GAL4/nopo*^*excl42*^	**0.00**	0.00	**0.00**	0.00	**0.00**	0.00	**0.17**	0.42	**0.12**	0.37	**12.46**	0.87	**0.78**	1.30	0.15	NS
*w*^*1118*^*/+; GMR-GAL4/nopo*^*Z1447*^	**0.00**	0.00	**0.00**	0.00	**0.00**	0.00	**0.05**	0.34	**0.09**	0.34	**12.07**	0.55	**0.39**	0.79	0.09	NS
*w*^*1118*^*/+; GMR-GAL4/+; park*^*1 / +*^	**0.00**	0.00	**0.00**	0.00	**0.00**	0.00	**0.08**	0.32	**0.07**	0.30	**12.07**	0.38	**0.27**	0.88	0.10	NS
*w*^*1118*^*/+; GMR-GAL4/+; park*^*∆21/+*^	**0.00**	0.00	**0.00**	0.00	**0.00**	0.00	**0.11**	0.31	**0.07**	0.25	**12.07**	0.37	**0.33**	0.65	0.08	NS
**Group 2:**	** **	** **	** **	** **	** **	** **	** **	** **	** **	** **	** **	** **			** **	** **
*w*^*1118*^*/+; GMR-GAL4/+; UAS-cindr*^*RNAi 2*.*21*^*/+*	**0.04**	0.20	**0.17**	0.38	**0.05**	0.23	**0.59**	0.62	**1.81**	0.82	**10.47**	1.06	**4.20**	1.87	0.22	-
*w*^*1118*^*/+; GMR-GAL4/nopo*^*excl42*^*; UAS-cindr*^*RNAi 2*.*21*^*/+*	**0.07**	0.25	**0.37**	0.71	**0.04**	0.20	**0.83**	0.79	**2.04**	0.73	**12.44**	1.55	**4.64**	1.93	0.22	0.15884
*w*^*1118*^*/+; GMR-GAL4/nopo*^*Z1447*^*; UAS-cindr*^*RNAi 2*.*21*^*/+*	**0.01**	0.12	**0.31**	0.61	**0.12**	0.33	**1.16**	0.75	**2.23**	0.71	**11.07**	1.53	**5.33**	1.99	0.23	0.00049
*w*^*1118*^*/+; GMR-GAL4/+; UAS-cindr*^*RNAi 2*.*21*^*/park*^*1*^	**0.00**	0.00	**0.15**	0.39	**0.00**	0.00	**0.55**	0.62	**1.51**	0.89	**10.52**	1.02	**3.75**	1.62	0.19	0.11521
*w*^*1118*^*/+; GMR-GAL4/+; UAS-cindr*^*RNAi 2*.*21*^*/park*^*∆21*^	**0.00**	0.00	**0.32**	0.62	**0.07**	0.25	**0.75**	0.74	**1.56**	0.86	**11.43**	1.11	**3.66**	1.92	0.22	0.08381
**Group 3:**	** **	** **	** **	** **	** **	** **	** **	** **	** **	** **	** **	** **			** **	** **
*w*^*1118*^*/+; GMR-GAL4/UAS-lacZ*	**0.00**	0.00	**0.00**	0.00	**0.00**	0.00	**0.09**	0.29	**0.04**	0.20	**12.22**	0.43	**0.37**	0.61	0.07	-
*w*^*1118*^*/+; GMR-GAL4/UAS-nopo*	**0.00**	0.00	**0.00**	0.00	**0.00**	0.00	**0.04**	0.20	**0.03**	0.16	**12.28**	0.56	**0.35**	0.65	0.07	NS
*w*^*1118*^*/+; GMR-GAL4/UAS-park*	**0.00**	0.00	**0.00**	0.00	**0.00**	0.00	**0.09**	0.29	**0.16**	0.37	**12.03**	0.43	**0.44**	0.78	0.09	NS
*w*^*1118*^*/+; GMR-GAL4/UAS-traf6*	**0.00**	0.00	**0.00**	0.00	**0.01**	0.12	**0.08**	0.27	**0.08**	0.27	**12.26**	0.48	**0.43**	0.79	0.09	NS
**Group 4:**	** **	** **	** **	** **	** **	** **	** **	** **	** **	** **	** **	** **			** **	** **
*w*^*1118*^*/+; GMR-GAL4/UAS-lacZ; UAS-cindr*^*RNAi 2*.*21*^*/+*	**0.05**	0.23	**0.31**	0.52	**0.03**	0.16	**0.60**	0.72	**1.93**	0.86	**10.97**	0.90	**4.09**	1.83	0.21	-
*w*^*1118*^*/+; GMR-GAL4/UAS-nopo; UAS-cindr*^*RNAi 2*.*21*^*/+*	**0.00**	0.00	**0.07**	0.25	**0.01**	0.12	**0.27**	0.47	**0.72**	0.76	**12.05**	0.84	**1.61**	1.43	0.17	4.38 x 10^−16^
*w*^*1118*^*/+; GMR-GAL4/UAS-park; UAS-cindr*^*RNAi 2*.*21*^*/+*	**0.03**	0.16	**0.09**	0.29	**0.00**	0.00	**0.32**	0.57	**1.04**	0.83	**11.15**	0.78	**2.39**	1.66	0.19	1.79 x 10^−8^
*w*^*1118*^*/+; GMR-GAL4/UAS-traf6; UAS-cindr*^*RNAi 2*.*21*^*/+*	**0.03**	0.16	**0.12**	0.37	**0.03**	0.16	**0.40**	0.55	**1.73**	0.89	**11.03**	1.00	**3.35**	1.66	0.19	0.01114

1. 75 hexagonal data points were analyzed per genotype.

Hexagonal data points were determined as described in Johnson and Cagan, 2009. Briefly, patterning errors included

2. Defects in the orientation and number of cone cells.

3. Defects in the number, relative size and shape of primary pigment cells.

4. Incorrect orientation of the ommatial core with respect to the dorsal-ventral axis of the eye.

5. Defects in the position or number of bristles.

6. Missing tertiary pigment cells.

7. Number of missing or excess interommatidial cells (12 interommatidial cells lie within each data point in wild-type retinas).

8. The mean mispatterning score is the mean number of total errors observed per hexagonal field. SD = standard deviation; SE = standard error.

9. Mispatterning scores were compared using students' t-tests to determine statistical significance. For Group 1, datasets were compared to w1118/+; GMR-GAL4/+. For Group 2, datasets were compared to w1118/+; GMR-GAL4/+; UAS-cindr RNAi2.21/+. For Group 3. datasets were compared to w1118/+; GMR-GAL4/UAS-lacZ. For Group 4, datasets were compared to w1118/+; GMR-GAL4/UAS-lacZ; UAS-cindrRNAi2.21/+. Datasets were significantly different at the 1% confidence level if p<0.01, the 5% confidence level if p<0.05 and the 10% level if p<0.1. NS = not significant.

## Discussion

Many signals and cell behaviors integrate to pattern complex epithelia. In this screen, we have identified 36 E3 ligases and 4 Cullins that interact with the adaptor protein Cindr, which is required for these processes (Table 2). Few of these E3s/Cullins have been characterized and the roles of most of these in epithelia are unexplored. Deciphering the substrates of the E3 ligases and the conserved cell behaviors that they modify will be an important next step in understanding their contribution to epithelial patterning.

Amongst those loci identified in our screen were a set encoding E3 ligases that had previously been implicated in modifying JNK activity (Fig 2). These E3s modified *cindr*^*RNAi*^-induced patterning defects that were evident in the errant arrangement of cells in the pupal eye and reflected in the disordered arrangement of lens facets in the adult. Cindr is required to inhibit JNK activity in the developing *Drosophila* wing epithelium [16]. Hence identifying *nopo*, *parkin* and *Traf6* in our screen likely reflects that Cindr-JNK interactions are important for the correct development of most epithelia. However, since many E3 ligases regulate multiple proteins, it is possible that Nopo, Parkin or Traf6 have targets besides components of the JNK cascade. Over-expression of these proteins did not disrupt the eye (Fig 4E, 4I and 4M) and investigations of loss-of-function phenotypes are required to clarify whether these E3 ligases contribute to JNK-independent processes that pattern epithelia.

It is intriguing that genetic manipulations that potentially increased JNK activity (ectopic *bsk* and *slpr* expression, mutations in *puc*) as well as those that perturbed JNK (mutations in *bsk*, expression of *puc*) enhanced mis-patterning of the adult *GMR>cindr*^*RNAi*^ fly eye. Solving this anomaly will require investigation of the cell behaviors regulated by JNK during larval and pupal eye development. However, our data are less surprising if one considers the effect of specific cell behaviors that converge to organize the eye. For example, during pupal development, local cell movements rearrange interommatidial cells to generate the honeycomb lattice [77] and too much cell movement, as well as too little, can impede patterning to generate adult eyes that appear similarly disordered. Hence, whilst the adult *Drosophila* eye provides an excellent model for genetic screens such as the one described in this manuscript, further investigations may be essential to pinpoint the cell behaviors that generate adult eye phenotypes.

Our screen did not include all E3 ligases encoded in the *Drosophila* genome and some of the alleles used may not have disrupted gene expression sufficiently to modify the cell behaviors responsible for mis-patterning of the *GMR>cindr*^*RNAi2*^ eye. Nonetheless, we have identified a large number of E3 ligases and Cullins that potentially function with Cindr to modify the cytoskeleton, adhesion or signaling as the eye epithelium is organized [13–16]. Due to the high degree of conservation between *Drosophila* and vertebrates, the orthologs of these E3 ligases and Cullins are likely to modify processes regulated by CD2AP and CIN85, the vertebrate orthologs of Cindr [78–83].

## Supporting information

S1 Table List of experimentally determined or predicted E3 ligase proteins (excludes SKP1 and F-box domain proteins). (DOCX)
