# Supplementary material for: A screen for E3 ubiquitination ligases that genetically interact with the adaptor protein Cindr during Drosophila eye patterning
Source: PLoS One. 2017 Nov 8;12(11):e0187571. doi: 10.1371/journal.pone.0187571 (PMC5678704; doi:10.1371/journal.pone.0187571)
Supplement: S1 Table — (DOCX) [file pone.0187571.s001.docx]

| **S1 Table: List of experimentally determined or predicted E3 ligase and Cullin proteins (excludes SKP1 and F-box domain proteins)** | | | |
| --- | --- | --- | --- |
| **Known/**  **predicted domain class^1.^** | **CG number** | **Gene symbol** | **Included in screen? ^2.^** |
| Cullin | CG11261 |  | yes |
| Cullin | CG11861 | *Cul3* | yes |
| Cullin | CG1401 | *Cul5* | yes |
| Cullin | CG1512 | *Cul2* | yes |
| Cullin | CG1877 | *Cul1* | yes |
| Cullin | CG8711 | *Cul4* | yes |
| Goliath | CG10277 | *godzilla* | excluded, reported role in endosome recycling [1]. |
| Goliath | CG2679 | *gol* | excluded, reported role in endosome recycling [1]. |
| HECT | CG11734 | *HERC2* | excluded, no suitable alleles available |
| HECT | CG3356 |  | excluded, no suitable alleles available |
| HECT | CG4238 |  | yes |
| HECT | CG4244 | *Su(dx)* | excluded, reported role in Notch signaling [2] |
| HECT | CG42574 | *ctrip* | yes |
| HECT | CG42797 |  | excluded, no suitable alleles available |
| HECT | CG4943 | *Smurf*  *(lack)* | excluded, reported role in Dpp, Hh and Hippo signaling [3-6] |
| HECT | CG5087 |  | excluded, no suitable alleles available |
| HECT | CG5604 | *Ufd4* | excluded, no suitable alleles available |
| HECT | CG6190 | *Ube3a* | yes |
| HECT | CG7555 | *Nedd4* | excluded, reported role in endocytosis of proteins including Notch [7, 8] |
| HECT | CG8184 |  | yes |
| HECT | CG9153 | *Sherpa* | yes |
| HECT | CG9484 | *hyd* | yes |
| Inhibitor of apoptosis | CG12284 | *Diap1* | excluded, regulator of cell death |
| Inhibitor of apoptosis | CG8293 | *Diap2* | excluded, regulator of cell death |
| RING | CG10144 | *Vps8* | excluded, no suitable alleles available, reported role in endosome trafficking [9] |
| RING | CG10263 | *Hakai* | yes |
| RING | CG10523 | *park* | yes |
| RING | CG10542 | *Bre1* | excluded, reported role in histone H2B modification and Notch signaling [10] |
| RING | CG10761 |  | excluded, predicted to reside in ER or mitochondria |
| RING | CG10916 |  | yes |
| RING | CG10916 | *Traf6* | yes |
| RING | CG10981 | *dgrn* | yes |
| RING | CG11281 | *snky* | excluded, no suitable alleles available |
| RING | CG11329 | *Nse1* | yes |
| RING | CG1134 | *Mul1* | excluded, mitochondrial function [11] |
| RING | CG11360 |  | excluded, no suitable alleles available |
| RING | CG11414 |  | excluded, predicted role in nucleus & mitosis |
| RING | CG11982 |  | excluded, implicated in auto-ubiquitination |
| RING | CG11988 | *neur* | excluded, reported role in Notch signaling [12]. |
| RING | CG12099 |  | excluded, implicated in auto-ubiquitination |
| RING | CG12200 |  | excluded, no suitable alleles available |
| RING | CG12477 |  | yes |
| RING | CG12489 | *dnr1* | yes |
| RING | CG13025 |  | yes |
| RING | CG13030 | *sinah* | yes |
| RING | CG1317 |  | excluded, predicted to reside in ER |
| RING | CG13344 |  | yes |
| RING | CG13442 |  | excluded, predicted ER/lysosome protein |
| RING | CG13481 |  | excluded, no suitable alleles available |
| RING | CG14472 | *poe* | yes |
| RING | CG13605 |  | yes |
| RING | CG14435 |  | excluded, no suitable alleles available |
| RING | CG14983 |  | excluded, no suitable alleles available |
| RING | CG15011 |  | excluded, predicted to reside in nucleus |
| RING | CG15104 | *Topors* | yes |
| RING | CG15141 |  | yes |
| RING | CG15150 | *elfless* | excluded, expressed in germline, nuclear protein [13] |
| RING | CG15439 |  | yes |
| RING | CG15814 |  | excluded, no suitable alleles available |
| RING | CG16781 |  | excluded, no suitable alleles available |
| RING | CG16807 | *roq* | yes |
| RING | CG16947 |  | yes |
| RING | CG17019 |  | excluded, no suitable alleles available |
| RING | CG17033 | *elgi* | excluded, no suitable alleles available |
| RING | CG17048 |  | excluded, no suitable alleles available |
| RING | CG17260 |  | excluded, no suitable alleles available |
| RING | CG17329 |  | excluded, no suitable alleles available |
| RING | CG17492 | *mib2* | yes |
| RING | CG17717 |  | excluded, no suitable alleles available |
| RING | CG17721 |  | yes |
| RING | CG17991 |  | excluded, no suitable alleles available |
| RING | CG18028 | *lt* | excluded, reported role in endosome/lysosome trafficking [9] |
| RING | CG1815 |  | yes |
| RING | CG1909 |  | yes |
| RING | CG1937 | *sip3* | excluded, ER membrane protein, reported role in ER-associated degradation [14] |
| RING | CG2304 | *Trc8* | excluded, predicted ER protein that interacts with lipid and protein biosynthetic pathways [15] |
| RING | CG2617 |  | excluded, no suitable alleles available |
| RING | CG2681 |  | excluded, no suitable alleles available |
| RING | CG2682 | *d4* | excluded, no suitable alleles available |
| RING | CG2709 | *vilya* | excluded, nuclear, reported role in meiosis [16, 17] |
| RING | CG3093 | *dor* | excluded, reported roles in endosome/lysosome trafficking [18, 19] |
| RING | CG31053 |  | excluded, no suitable alleles available |
| RING | CG31716 | *Cnot4* | excluded, no suitable alleles available |
| RING | CG31807 |  | excluded, no suitable alleles available |
| RING | CG32210 | *Ltn1* | yes |
| RING | CG3231 | *snama* | excluded, predicted nuclear protein |
| RING | CG32350 | *Vps11* | excluded, no suitable alleles available, reported role in endosome trafficking [9] |
| RING | CG32369 |  | yes |
| RING | CG3241 | *Msl-2* | excluded, nuclear protein, component of the male specific lethal complex [20] |
| RING | CG32486 |  | yes |
| RING | CG32581 |  | yes |
| RING | CG32592 | *hiw* | yes |
| RING | CG32847 |  | excluded, no suitable alleles available |
| RING | CG32850 |  | excluded, no suitable alleles available |
| RING | CG33552 |  | excluded, predicted to reside in nucleus |
| RING | CG34289 |  | excluded, no suitable alleles available |
| RING | CG34308 |  | excluded, no suitable alleles available |
| RING | CG34375 |  | excluded, predicted to reside in nucleus |
| RING | CG34440 | *LmgA* | excluded, subunit of the APC |
| RING | CG3639 | *Pex12* | excluded, peroxisomal protein [21] |
| RING | CG3647 | *stc* | excluded, predicted nuclear protein |
| RING | CG3886 | *Psc* | excluded, nuclear protein, reported role in histone modification, component of Polycomb repressive complex 1 [22] |
| RING | CG3905 | *Su(z)2* | excluded, nuclear protein, reported role in chromatin regulation, a polycomb group protein [23] |
| RING | CG3929 | *dx* | excluded, reported role in Notch [24, 25] |
| RING | CG4030 | *Rbpn-5* | excluded, reported role in endosomal dynamics [26] |
| RING | CG4080 |  | yes |
| RING | CG4195 | *l(3)73Ah* | excluded, predicted to reside in nucleus |
| RING | CG42593 | *Ubr3* | excluded, reported role in apoptosis [27] |
| RING | CG43726 | *qin* | excluded, nuclear protein, reported role in piRNA regulation [28, 29] |
| RING | CG4620 | *unk* | excluded, reported role in photoreceptor differentiation [30] |
| RING | CG4909 | *POSH* | yes |
| RING | CG4973 | *mdlc* | excluded, no suitable alleles available |
| RING | CG5071 |  | excluded, no suitable alleles available |
| RING | CG5140 | *nopo* | yes |
| RING | CG5347 |  | excluded, no suitable alleles available |
| RING | CG5382 |  | excluded, no suitable alleles available |
| RING | CG5555 |  | yes |
| RING | CG5591 | *Lpt* | excluded, reported roles in chromatin/histone modification and transcription [31-33] |
| RING | CG5595 | *Sce* | excluded, nuclear protein, reported role in histone modification, component of Polycomb repressive complex 1 [22] |
| RING | CG5841 | *mib1* | excluded, but should have excluded, reported role in Notch signaling [12, 34, 35] |
| RING | CG6688 |  | excluded, predicted to reside in nucleus |
| RING | CG6752 |  | yes |
| RING | CG6923 |  | yes |
| RING | CG7037 | *Cbl* | excluded, reported role in RTK and Notch signaling [36-38] |
| RING | CG7081 | *Pex2* | excluded, peroxisomal protein [21] |
| RING | CG7184 | *Mkrn1* | yes |
| RING | CG7376 |  | yes |
| RING | CG7614 | *Mat1* | excluded, component of the CDK-activating kinase complex [39] |
| RING | CG7694 |  | yes |
| RING | CG7864 | *Pex10* | excluded, peroxisomal protein [21] |
| RING | CG8103 | *Mi-2* | excluded, nuclear, reported role in chromatin remodeling [40-42] |
| RING | CG8141 |  | excluded, no suitable alleles available |
| RING | CG8651 | *trx* | excluded, nuclear protein, reported role in chromatin regulation [43-45] |
| RING | CG8786 |  | yes |
| RING | CG8910 |  | yes |
| RING | CG8974 |  | yes |
| RING | CG9014 |  | excluded, no suitable alleles available |
| RING | CG9086 | *Ubr1* | yes |
| RING | CG9381 | *mura* | yes |
| RING | CG9855 |  | exclude, predicted to reside in ER or mitochondria |
| RING | CG9941 |  | yes |
| RING | CG9949 | *sina* | yes |
| RING-between-RING | CG11321 | *LUBEL* | yes |
| RING-between-RING | CG12362 |  | yes |
| RING-between-RING | CG33144 |  | yes |
| RING-between-RING | CG5659 | *ari-1* | yes |
| RING-between-RING | CG5709 | *ari-2* | yes |
| ROC | CG16982 | *Roc1a* | yes |
| ROC | CG16988 | *Roc1b* | yes |
| ROC | CG8998 | *Roc2* | yes |
| TRIM | CG12218 | *mei-P26* | excluded, reported role in germline [46] |
| TRIM | CG15105 | *tn* | excluded, Z-band protein ** |
| TRIM | CG31721 | *Trim9* | yes |
| TRIM | CG5206 | *bon* | excluded, resides in nucleus |
| TRIM | CG8419 |  | yes |
| U-box | CG2218 |  | yes |
| U-box | CG5203 | *STUB1* | excluded, no suitable alleles available |
| U-box | CG5519 | *Prp19* | yes |
| U-box | CG6179 |  | yes |
| U-box | CG7747 |  | yes |
| U-box | CG9934 |  | yes |
| 1. Classification according to <http://flybase.org/reports/FBgg0000069.html>, <http://flybase.org/reports/FBgg0000128.html>, and <http://flybase.org/reports/FBgg0000131.html> 2. Brief reason for exclusion; references provided in attached list. | | | |

References

1. Yamazaki Y, Schonherr C, Varshney GK, Dogru M, Hallberg B, Palmer RH. Goliath family E3 ligases regulate the recycling endosome pathway via VAMP3 ubiquitylation. EMBO J. 2013;32(4):524-37. doi: 10.1038/emboj.2013.1. PubMed PMID: 23353890; PubMed Central PMCID: PMCPMC3579141.

2. Fostier M, Evans DA, Artavanis-Tsakonas S, Baron M. Genetic characterization of the Drosophila melanogaster Suppressor of deltex gene: A regulator of notch signaling. Genetics. 1998;150(4):1477-85. PubMed PMID: 9832525; PubMed Central PMCID: PMCPMC1460411.

3. Podos SD, Hanson KK, Wang YC, Ferguson EL. The DSmurf ubiquitin-protein ligase restricts BMP signaling spatially and temporally during Drosophila embryogenesis. Dev Cell. 2001;1(4):567-78. PubMed PMID: 11703946.

4. Liang YY, Lin X, Liang M, Brunicardi FC, ten Dijke P, Chen Z, et al. dSmurf selectively degrades decapentaplegic-activated MAD, and its overexpression disrupts imaginal disc development. J Biol Chem. 2003;278(29):26307-10. doi: 10.1074/jbc.C300028200. PubMed PMID: 12754252.

5. Cao L, Wang P, Gao Y, Lin X, Wang F, Wu S. Ubiquitin E3 ligase dSmurf is essential for Wts protein turnover and Hippo signaling. Biochem Biophys Res Commun. 2014;454(1):167-71. doi: 10.1016/j.bbrc.2014.10.058. PubMed PMID: 25450375.

6. Huang S, Zhang Z, Zhang C, Lv X, Zheng X, Chen Z, et al. Activation of Smurf E3 ligase promoted by smoothened regulates hedgehog signaling through targeting patched turnover. PLoS Biol. 2013;11(11):e1001721. doi: 10.1371/journal.pbio.1001721. PubMed PMID: 24302888; PubMed Central PMCID: PMCPMC3841102.

7. Ing B, Shteiman-Kotler A, Castelli M, Henry P, Pak Y, Stewart B, et al. Regulation of Commissureless by the ubiquitin ligase DNedd4 is required for neuromuscular synaptogenesis in Drosophila melanogaster. Mol Cell Biol. 2007;27(2):481-96. doi: 10.1128/MCB.00463-06. PubMed PMID: 17074801; PubMed Central PMCID: PMCPMC1800811.

8. Sakata T, Sakaguchi H, Tsuda L, Higashitani A, Aigaki T, Matsuno K, et al. Drosophila Nedd4 regulates endocytosis of notch and suppresses its ligand-independent activation. Curr Biol. 2004;14(24):2228-36. doi: 10.1016/j.cub.2004.12.028. PubMed PMID: 15620649.

9. Solinger JA, Spang A. Tethering complexes in the endocytic pathway: CORVET and HOPS. FEBS J. 2013;280(12):2743-57. doi: 10.1111/febs.12151. PubMed PMID: 23351085.

10. Bray S, Musisi H, Bienz M. Bre1 is required for Notch signaling and histone modification. Dev Cell. 2005;8(2):279-86. doi: 10.1016/j.devcel.2004.11.020. PubMed PMID: 15691768.

11. Yun J, Puri R, Yang H, Lizzio MA, Wu C, Sheng ZH, et al. MUL1 acts in parallel to the PINK1/parkin pathway in regulating mitofusin and compensates for loss of PINK1/parkin. Elife. 2014;3:e01958. doi: 10.7554/eLife.01958. PubMed PMID: 24898855; PubMed Central PMCID: PMCPMC4044952.

12. Wang W, Struhl G. Distinct roles for Mind bomb, Neuralized and Epsin in mediating DSL endocytosis and signaling in Drosophila. Development. 2005;132(12):2883-94. doi: 10.1242/dev.01860. PubMed PMID: 15930117.

13. Caldwell JC, Joiner ML, Sivan-Loukianova E, Eberl DF. The role of the RING-finger protein Elfless in Drosophila spermatogenesis and apoptosis. Fly (Austin). 2008;2(6):269-79. PubMed PMID: 19077536; PubMed Central PMCID: PMCPMC2668719.

14. Kang MJ, Ryoo HD. Suppression of retinal degeneration in Drosophila by stimulation of ER-associated degradation. Proc Natl Acad Sci U S A. 2009;106(40):17043-8. doi: 10.1073/pnas.0905566106. PubMed PMID: 19805114; PubMed Central PMCID: PMCPMC2749843.

15. Lee JP, Brauweiler A, Rudolph M, Hooper JE, Drabkin HA, Gemmill RM. The TRC8 ubiquitin ligase is sterol regulated and interacts with lipid and protein biosynthetic pathways. Mol Cancer Res. 2010;8(1):93-106. doi: 10.1158/1541-7786.MCR-08-0491. PubMed PMID: 20068067; PubMed Central PMCID: PMCPMC3086825.

16. Collins KA, Callicoat JG, Lake CM, McClurken CM, Kohl KP, Hawley RS. A germline clone screen on the X chromosome reveals novel meiotic mutants in Drosophila melanogaster. G3 (Bethesda). 2012;2(11):1369-77. doi: 10.1534/g3.112.003723. PubMed PMID: 23173088; PubMed Central PMCID: PMCPMC3484667.

17. Lake CM, Nielsen RJ, Guo F, Unruh JR, Slaughter BD, Hawley RS. Vilya, a component of the recombination nodule, is required for meiotic double-strand break formation in Drosophila. Elife. 2015;4:e08287. doi: 10.7554/eLife.08287. PubMed PMID: 26452093; PubMed Central PMCID: PMCPMC4703084.

18. Sevrioukov EA, He JP, Moghrabi N, Sunio A, Kramer H. A role for the deep orange and carnation eye color genes in lysosomal delivery in Drosophila. Mol Cell. 1999;4(4):479-86. PubMed PMID: 10549280.

19. Pulipparacharuvil S, Akbar MA, Ray S, Sevrioukov EA, Haberman AS, Rohrer J, et al. Drosophila Vps16A is required for trafficking to lysosomes and biogenesis of pigment granules. J Cell Sci. 2005;118(Pt 16):3663-73. doi: 10.1242/jcs.02502. PubMed PMID: 16046475.

20. Hallacli E, Lipp M, Georgiev P, Spielman C, Cusack S, Akhtar A, et al. Msl1-mediated dimerization of the dosage compensation complex is essential for male X-chromosome regulation in Drosophila. Mol Cell. 2012;48(4):587-600. doi: 10.1016/j.molcel.2012.09.014. PubMed PMID: 23084835.

21. Faust JE, Verma A, Peng C, McNew JA. An inventory of peroxisomal proteins and pathways in Drosophila melanogaster. Traffic. 2012;13(10):1378-92. doi: 10.1111/j.1600-0854.2012.01393.x. PubMed PMID: 22758915; PubMed Central PMCID: PMCPMC3443258.

22. Lagarou A, Mohd-Sarip A, Moshkin YM, Chalkley GE, Bezstarosti K, Demmers JA, et al. dKDM2 couples histone H2A ubiquitylation to histone H3 demethylation during Polycomb group silencing. Genes Dev. 2008;22(20):2799-810. doi: 10.1101/gad.484208. PubMed PMID: 18923078; PubMed Central PMCID: PMCPMC2569881.

23. Lo SM, Ahuja NK, Francis NJ. Polycomb group protein Suppressor 2 of zeste is a functional homolog of Posterior Sex Combs. Mol Cell Biol. 2009;29(2):515-25. doi: 10.1128/MCB.01044-08. PubMed PMID: 18981224; PubMed Central PMCID: PMCPMC2612506.

24. Fuwa TJ, Hori K, Sasamura T, Higgs J, Baron M, Matsuno K. The first deltex null mutant indicates tissue-specific deltex-dependent Notch signaling in Drosophila. Mol Genet Genomics. 2006;275(3):251-63. doi: 10.1007/s00438-005-0087-3. PubMed PMID: 16395579.

25. Wilkin M, Tongngok P, Gensch N, Clemence S, Motoki M, Yamada K, et al. Drosophila HOPS and AP-3 complex genes are required for a Deltex-regulated activation of notch in the endosomal trafficking pathway. Dev Cell. 2008;15(5):762-72. doi: 10.1016/j.devcel.2008.09.002. PubMed PMID: 19000840.

26. Thomas C, Strutt D. Rabaptin-5 and Rabex-5 are neoplastic tumour suppressor genes that interact to modulate Rab5 dynamics in Drosophila melanogaster. Dev Biol. 2014;385(1):107-21. doi: 10.1016/j.ydbio.2013.09.029. PubMed PMID: 24104056; PubMed Central PMCID: PMCPMC3858806.

27. Huang Q, Tang X, Wang G, Fan Y, Ray L, Bergmann A, et al. Ubr3 E3 ligase regulates apoptosis by controlling the activity of DIAP1 in Drosophila. Cell Death Differ. 2014;21(12):1961-70. doi: 10.1038/cdd.2014.115. PubMed PMID: 25146930; PubMed Central PMCID: PMCPMC4227149.

28. Anand A, Kai T. The tudor domain protein kumo is required to assemble the nuage and to generate germline piRNAs in Drosophila. EMBO J. 2012;31(4):870-82. doi: 10.1038/emboj.2011.449. PubMed PMID: 22157814; PubMed Central PMCID: PMCPMC3280549.

29. Zhang Z, Koppetsch BS, Wang J, Tipping C, Weng Z, Theurkauf WE, et al. Antisense piRNA amplification, but not piRNA production or nuage assembly, requires the Tudor-domain protein Qin. EMBO J. 2014;33(6):536-9. doi: 10.1002/embj.201384895. PubMed PMID: 24652836; PubMed Central PMCID: PMCPMC3989648.

30. Avet-Rochex A, Carvajal N, Christoforou CP, Yeung K, Maierbrugger KT, Hobbs C, et al. Unkempt is negatively regulated by mTOR and uncouples neuronal differentiation from growth control. PLoS Genet. 2014;10(9):e1004624. doi: 10.1371/journal.pgen.1004624. PubMed PMID: 25210733; PubMed Central PMCID: PMCPMC4161320.

31. Chauhan C, Zraly CB, Dingwall AK. The Drosophila COMPASS-like Cmi-Trr coactivator complex regulates dpp/BMP signaling in pattern formation. Dev Biol. 2013;380(2):185-98. doi: 10.1016/j.ydbio.2013.05.018. PubMed PMID: 23707261.

32. Chauhan C, Zraly CB, Parilla M, Diaz MO, Dingwall AK. Histone recognition and nuclear receptor co-activator functions of Drosophila cara mitad, a homolog of the N-terminal portion of mammalian MLL2 and MLL3. Development. 2012;139(11):1997-2008. doi: 10.1242/dev.076687. PubMed PMID: 22569554; PubMed Central PMCID: PMCPMC3347691.

33. Mohan M, Herz HM, Smith ER, Zhang Y, Jackson J, Washburn MP, et al. The COMPASS family of H3K4 methylases in Drosophila. Mol Cell Biol. 2011;31(21):4310-8. doi: 10.1128/MCB.06092-11. PubMed PMID: 21875999; PubMed Central PMCID: PMCPMC3209330.

34. Le Borgne R, Remaud S, Hamel S, Schweisguth F. Two distinct E3 ubiquitin ligases have complementary functions in the regulation of delta and serrate signaling in Drosophila. PLoS Biol. 2005;3(4):e96. doi: 10.1371/journal.pbio.0030096. PubMed PMID: 15760269; PubMed Central PMCID: PMCPMC1064853.

35. Pitsouli C, Delidakis C. The interplay between DSL proteins and ubiquitin ligases in Notch signaling. Development. 2005;132(18):4041-50. doi: 10.1242/dev.01979. PubMed PMID: 16093323.

36. Hime GR, Dhungat MP, Ng A, Bowtell DD. D-Cbl, the Drosophila homologue of the c-Cbl proto-oncogene, interacts with the Drosophila EGF receptor in vivo, despite lacking C-terminal adaptor binding sites. Oncogene. 1997;14(22):2709-19. doi: 10.1038/sj.onc.1201223. PubMed PMID: 9178769.

37. Meisner H, Daga A, Buxton J, Fernandez B, Chawla A, Banerjee U, et al. Interactions of Drosophila Cbl with epidermal growth factor receptors and role of Cbl in R7 photoreceptor cell development. Mol Cell Biol. 1997;17(4):2217-25. PubMed PMID: 9121472; PubMed Central PMCID: PMCPMC232071.

38. Wang Y, Chen Z, Bergmann A. Regulation of EGFR and Notch signaling by distinct isoforms of D-cbl during Drosophila development. Dev Biol. 2010;342(1):1-10. doi: 10.1016/j.ydbio.2010.03.005. PubMed PMID: 20302857; PubMed Central PMCID: PMCPMC2866751.

39. Larochelle S, Chen J, Knights R, Pandur J, Morcillo P, Erdjument-Bromage H, et al. T-loop phosphorylation stabilizes the CDK7-cyclin H-MAT1 complex in vivo and regulates its CTD kinase activity. EMBO J. 2001;20(14):3749-59. doi: 10.1093/emboj/20.14.3749. PubMed PMID: 11447116; PubMed Central PMCID: PMCPMC125544.

40. Bouazoune K, Brehm A. dMi-2 chromatin binding and remodeling activities are regulated by dCK2 phosphorylation. J Biol Chem. 2005;280(51):41912-20. doi: 10.1074/jbc.M507084200. PubMed PMID: 16223721.

41. Bouazoune K, Mitterweger A, Langst G, Imhof A, Akhtar A, Becker PB, et al. The dMi-2 chromodomains are DNA binding modules important for ATP-dependent nucleosome mobilization. EMBO J. 2002;21(10):2430-40. doi: 10.1093/emboj/21.10.2430. PubMed PMID: 12006495; PubMed Central PMCID: PMCPMC125999.

42. Murawska M, Kunert N, van Vugt J, Langst G, Kremmer E, Logie C, et al. dCHD3, a novel ATP-dependent chromatin remodeler associated with sites of active transcription. Mol Cell Biol. 2008;28(8):2745-57. doi: 10.1128/MCB.01839-07. PubMed PMID: 18250149; PubMed Central PMCID: PMCPMC2293103.

43. Joanis V, Lloyd VK. Genomic imprinting in Drosophila is maintained by the products of Suppressor of variegation and trithorax group, but not Polycomb group, genes. Mol Genet Genomics. 2002;268(1):103-12. doi: 10.1007/s00438-002-0731-0. PubMed PMID: 12242505.

44. Papoulas O, Beek SJ, Moseley SL, McCallum CM, Sarte M, Shearn A, et al. The Drosophila trithorax group proteins BRM, ASH1 and ASH2 are subunits of distinct protein complexes. Development. 1998;125(20):3955-66. PubMed PMID: 9735357.

45. Petruk S, Sedkov Y, Smith S, Tillib S, Kraevski V, Nakamura T, et al. Trithorax and dCBP acting in a complex to maintain expression of a homeotic gene. Science. 2001;294(5545):1331-4. doi: 10.1126/science.1065683. PubMed PMID: 11701926.

46. Page SL, McKim KS, Deneen B, Van Hook TL, Hawley RS. Genetic studies of mei-P26 reveal a link between the processes that control germ cell proliferation in both sexes and those that control meiotic exchange in Drosophila. Genetics. 2000;155(4):1757-72. PubMed PMID: 10924472; PubMed Central PMCID: PMCPMC1461182.
